# Corporate social responsibility and job applicant attraction: A moderated-mediation model

**DOI:** 10.1371/journal.pone.0260125

**Published:** 2022-03-03

**Authors:** Hong-yan Wang, Zhi-Xia Chen

**Affiliations:** 1 College of Economics and Management, Hubei Polytechnic University, Huangshi, Hubei, People’s Republic of China; 2 College of Public Administration, Huazhong University of Science and Technology, Wuhan, Hubei, People’s Republic of China; Hong Kong Polytechnic University, HONG KONG

## Abstract

Based on the social identity theory, this study investigates the mediation and moderation mechanism of CSR on job applicant attraction. A total of 395 job seekers are recruited to join in the experiment survey. The results indicate that job seekers’ perceptions of CSR positively relate to job applicant attraction, employer reputation and expected pride mediate this relationship, respectively, and the serial mediating role of employer reputation and then expected pride in the relationship between CSR and job applicant attraction. Additionally, the findings show that job applicants’ materialism orientation plays a moderating role in the indirect effect of CSR on job applicant attraction via expected pride, but the moderating effect of job seekers’ materialism orientation in the indirect effect of CSR on job applicant attraction via employer reputation is not statistically significant. These findings enrich the new culture-driven evidence on the impacting mechanism of CSR on job applicant’s attitude and provide valuable insight into how CSR motivates job applicant attraction.

## Introduction

Corporate social responsibility(CSR) refers to the organization’s policies and actions to advance or promote some social good and stakeholders’ expectations beyond the organization’ and shareholders’ immediate interests and the mandatory law [1,2]. CSR has become a part of the core business approach that aims for sustainable development by providing economic, social, and environmental benefits to all stakeholders [3]. Research shows that better CSR activities help organizations acquire more resources, and earn optimal profits [4], and enhance job applicant attractiveness [5–8]. However, extant studies mainly explore the direct influence of CSR on job applicant attraction from multi-dimensional perspectives of CSR, the underlying mechanisms by which a multitude of organizational implications related to CSR would lead to improvements in job applicant attraction is still not profoundly understood [6]. Signaling theory suggests that a firm’s CSR sends signals to prospective job applicants about what it would be like to work for a firm [9]. Thus, most research has explored CSR’s influence on job applicant attraction based on the signaling theory. For instance, Jones et al. has confirmed that the three signal-based mechanisms (job seekers’ anticipated pride, perceived value fit, and expected treatment) affect the relationship between corporate social performance and organizational attractiveness [5,6]. Joo et al. also has examined the relationship between CSR and organizational attractiveness among job applicants from the perspective of overall justice and attributed motives [7]. While many empirical studies have found evidence supporting the role of signal in job seekers’ attitudes and behaviors toward CSR, social identification seems to have a powerful effect on how job applicants read CSR activities. According to social identity theory, people derive emotional significance from perceiving a sense of oneness with the group they identify [10]. Therefore, job seekers who identify to a higher degree with the employer may be more prone to generate positive emotional reactions (e.g. employer reputation, expected pride) to employer actions, thereby impacting their job applicant intentions. Based on social identity theory, therefore, this study may discuss the mediation role of employer reputation and expected pride in the relationship between CSR and job applicant attraction.

Moreover, it is unclear whether the relationship between CSR activities and job applicant attraction is culture-free or whether cross-national differences exist. While the literature advocates that employee’s attitude to CSR activities is culture-driven [11], most related studies have been conducted in the Western context. Cross-country comparisons are generally lacking in eastern culture, particularly in emerging economies [12]. The core of Confucianism ethics as characteristic of the traditional Chinese culture is humaneness, which advocates people’s social obligations and ethical decision-making [13,14]. These cultural values drive people to believe that it is a good ethical practice for organizations and individuals to perform social responsibility [15,16], and they also take social responsibility as vital information to evaluate and distinguish organizations [17]. Thus the emphasis is on maintaining a perfect organizational impression through the implementation of CSR activities, thereby promoting job applicant attraction, which is incredibly realistic and necessary for Chinese employers.

Furthermore, it is generally considered that the job seeker has a desire for material possession that originates from survival strategies evolved among modern human ancestors [18]. Researchers have proved that materialism orientation is associated with an individual’s happiness [19–21] and the conception of material consumption [22,23], however, the moderating effect of materialism orientation in the relationship between CSR and job applicant attraction has not been discussed systematically. High materialism-oriented individuals often view material wealth as the root of life, happiness, and success [24]. When such an individual embarks upon accumulating wealth and achieving societal standards of status and material accomplishment, he/she essentially acts in ways that are primarily governed by upward social comparisons from outside the individual [20]. This comparison places individuals on a ’hedonic treadmill,’ whereby more and more frequent acquisition becomes necessary to satisfy the individual’s competitive appetite [18]. However, materialism impedes the need to feel autonomous by taking individuals closer to externally regulated wants (fame, status, appearance) and farther away from their core psychological needs [20]. Conversely, low materialism-oriented individuals have lower expectations of the results of the social comparison process than individuals with high materialism orientation and emphasize the pleasure and satisfaction of their psychological needs [24]. The process of job seeking involves evaluating of the satisfaction of psychological needs brought by the organization and the consideration of the actual material benefits from the employer. Moreover, with its rapid economic growth, China is currently undergoing drastic changes in its social structure and consumption views, the accelerated accumulation of wealth has extensively promoted the rise of materialism which sets actual life goals, builds self-esteem and forms beliefs about others [22]. Job seekers’ materialism orientation therefore, is an unavoidable topic for both managers and researchers focusing on talent attraction. Above all, we reason that the individual’s materialism values are the most relevant moderator in the relationship between CSR and job applicant attraction.

The present study adds to the existing body of literature in three main ways. Firstly, it empirically extends the study of the relationship between CSR and job applicant attraction in the emerging economic context and eastern culture, namely China. The existing literature advocates that the relationship between CSR and employee attitude is culture-driven [11], so we propose that Chinese job seekers respond positively to CSR practices due to high levels of humaneness in the Chinese Confucian culture. This view may diverge from scholars’ opinion that Chinese employees will respond less positively to CSR practices targeted towards external stakeholders than employees in the West due to decreased job security levels and high levels of in-group collectivism [11]. The ethical humaneness thought of Confucianism culture highly emphasizes people’s social obligation and advocates people’s ethical decision-making [13,14], which are consistent with the original intention of CSR [15,16]. Thus job seekers in China, influenced by the Confucian culture, may value CSR more when evaluating potential employers, and those that can better engage in CSR practice will be more attractive to them. Therefore, this study, based on the Chinese traditional culture perspective, puts forward a new interpretation in the (potential) employee’s response to CSR. Secondly, this paper systematically explores the internal psychological mechanism of CSR and organizations’ attractiveness among job seekers based on social identity theory, which is a response to Jones et al.’s [6] appeal for research on the psychological mechanisms of CSR affecting job applicant attraction. Additionally, while a growing body of evidence indicates that materialism plays an integral part in people’s responses to different facets of their environment [20,21,23], this study is the first to investigate how an individual’s materialism orientation influences the attraction of job seekers to CSR practices. Finally, while other investigators have considered the classic statistical approaches, this research adds an experimental methodology that can effectively identify the relationships among core variables and promote the diversity of the study methodology applied in this area. To sum up, the findings should be of benefit to domestic and foreign organizations operating in China, enabling them to determine where it is best to concentrate on CSR activities to maximize job applicant attraction.

## Theory and hypotheses

### Corporate social responsibility (CSR) and job applicant attraction

During the past decade, increasing academic interest in CSR has led to the development of different definitions and the proposition of several methodologies to measure stakeholder perceptions of CSR management strategy [25]. CSR has been proved to generate several benefits for companies, such as improving relationships with customers and other stakeholders [26], increasing employee organizational commitment [11], organizational identification, and organizational pride [27]. However, these researches have focused on formal employees rather than job seekers as potential formal employees, only a few have examined that employers can attract job seekers by transmitting information about CSR activities in the recruitment process [7,8]. Job applicant attraction refers to a job seeker’s positive attitude or overall impression of the organization or perception of the organization as an ideal group to establish a relationship [28]. In the labor market, job seekers have asymmetric information about prospective employers, and they search for information that signals what the potential employers to which are applying [29]. Among the various signals, job seekers may focus on salient and distinctive information about employers. CSR activities for the good of society may be valued as a critical signal for job applicants to evaluate potential employers [29], thus giving job seekers a sense of what conditions to expect after being hired. Once job seekers identify with the employer, they will intend to become organization members [6–8]. Particularly the Chinese millennial-aged job applicants deeply influenced by Confucian culture attach great importance to social obligations orientation, moral behavior [16], and the intrinsic and social aspects of work [15], thus they may prefer to the employer engaged in CSR practices. This study, therefore, believes that the better an organization’s CSR performance, the more attractive it will be to applicants, and the hypothesis is as follows:

Hypothesis H1: CSR is positively correlated with job applicant attraction.

### Perceived employer reputation as a mediator

Employer reputation is the perception of its values and activities by its major stakeholders, and indicates the status of an organization’s name and brand relative to competing organizations and adds value to a job beyond the attributes of the job itself (e.g., work content, pay) [30]. From the job applicants’ perspective, employer reputation refers to job applicants’ overall evaluation and perception of an organization as a potential employer [28]. It incorporates both the cognitive reputation and emotional reputation, specifically by delays in perfect high talent attraction, improving social, environmental, other public affairs, and so on [28,31,32]. Previous research related to employer reputation has been studied mainly in the marketing field [32]. Until recently, a growing body of literature discusses the role of employer reputation in job applicant attraction [28,31]. The research states that employer reputation is more strongly related to job application decisions than is general reputation [30] and an organization with a favorable employer reputation is more attractive to the higher-quality applicants than those with a negative employer reputation [27,28,33].

Moreover, papers relevant to this topic have shown that CSR is one of the most widely known sources of reputation information and has a profound impact on employer reputation [5,6,27,32], and organizational identification [34]. Although numerous studies have shown the positive role of CSR practices in the employer’s reputation, fewer frameworks are applying the link of CSR with employer reputation in the recruitment field. CSR activities are a function of organizational signals of value [12,27], which are crucial cues for job seekers to evaluate prospective employer reputation [5,7,30,33]. According to social identity theory, CSR activities disclosure to the public benefits job seekers, allowing them to evaluate the employer’s reputation, which may have spillover effects on an individual’s perceived social status and identity [33]. Particularly, a job is more attractive to job applicants when the businesses are socially responsible with a positive reputation than those with low reputation [28,33], because an organization with a favorable employer reputation can lead to jobseekers’ enhanced sense of self-identity if they are hired [7,30,31]. However, it has also been recognized that CSR may bring an underlying risk to reputation, thereby impairing an organization’s ability to recruit job seekers [33]. Thus we hypothesize that employer reputation is an essential mechanism that establishes the link between CSR and job applicant attraction.

Hypothesis H2: Employer reputation mediates the relationship between CSR and job applicant attraction.

### Expected pride as a mediator

Expected pride in this study refers to a kind of psychological satisfaction generated in an individual due to his/her organization membership [35]. Self-improvement is a universally endorsed principle of human beings, individuals are motivated to seek the pride of their group members, as pride can enhance their self-evaluation and send a signal of higher social status to others [32]. Social identity theory suggests that people’s employers are an essential part of their self-concept and social identity, and the act of choosing and accepting an employer highlights the volitional nature of the affiliation [35]. Choosing an employer through a self-categorization and identity process represents a job seeker’s personal value orientation, and joining a particular organization is concrete, public expression of a person’s value and ability [36]. CSR activities, as the strong evidence of high capability and resourcefulness [32], spillover effects on an individual’s perceived superiority of social status [33] and organizational identification [34]. For job seekers, being affiliated with a prestigious organization may instill a strong sense of pride [5], enhancing the job applicant attraction.

Hypothesis H3: Expected pride mediates the relationship between CSR and job applicant attraction.

### The relationship of employer reputation and expected pride

As employer reputation and expected pride have a positive relationship with job applicant attraction, respectively, it is reasonable to assume that these two constructs are associated with each other. Previous research has also found that employer reputation, as an important source of competitive advantage, boosts organization-based self-esteem, thereby affiliation with an employer widely known for its positive reputation is likely to instil pride in membership [30,35]. When job seekers believe that an organization has a positive reputation, they may expect to "bask in the reflected glory" of the organization’s social position [30]. Conversely, the employer with negative reputations can confer the negative attributes of an organization on a member, replacing pride with embarrassment and discomfort [30,33]. As such, it is reasonable to infer that higher levels of employer reputation, result in higher levels of expected pride, which further increases job applicant attraction.

Hypothesis H4: CSR is indirectly associated with job applicant attraction by employer reputation and then expected pride.

### Materialism as a moderator

As one of the hallmarks of modern societies, materialism is used to assess the importance attached by individuals to material possessions as a route to happiness, success, and centrality [22,24]. Materialism is generally defined as " individual differences in people’s endorsement of values, goals, and associated beliefs that center on the importance of acquiring money and possessions that convey status." [19, p.880] Individuals with a high materialism orientation have a hedonistic present temporal focus [37] and view the accumulation of wealth, the acquisition of possessions, and improvement in their social status as the happy center of their life, while those with a low level of materialism attach greater importance to spiritual enlightenment [24]. Some researchers have found that individual thought and behavior, such as well-being [20,21] and consumption behavior [22,23], are influenced by individual materialism-orientation. However, the influence of individual materialism-orientation on the relationship between CSR and job applicant attraction is less explored. It is believed that high-materialism values have natural ties with upward social comparison, and materialists’ understanding of self-value is based on comparison with others to obtain the sense of superiority, and they may give more weight to immediate material benefits (e.g., wealth, fame and success) [38]. Also, some researchers have indicated that CSR helps corporations to gain better corporate reputation, and better corporate reputation helps corporations acquire more resources, and earns optimal profits [39,40]. But for the job seekers with high materialism orientation, the translation of these benefits into present temporal possession may take a certain time. Therefore, they tend to place less value on CSR activities that are not directly linked with tangible and instant outcomes, and may be less concerned about employer reputation. Likewise, the expected pride of the individuals with high scoring materialistic orientation may derive mainly from the gained material superiority by social comparison rather than from the intrinsic inherent beliefs elicited by employer’s CSR practices. In contrast, the job applicants with low-materialistic orientation tend to place importance on intangible spiritual goals, and it is possible that they have a preference for choosing these organizations which attach great importance to social responsibility practices. In their eyes, the positive intangible spiritual enlightenment benefit from CSR practice maybe more attractive than the instant material outcomes, and they will be inclined to give more importance to the employer reputation and expected pride from CSR activities. Therefore, this study proposes the following hypotheses:

Hypothesis H5a: the level of materialism orientation moderates the relationship between CSR and employer reputation such that the relationship is stronger for job applicants with a low materialistic orientation than for those with a high materialism orientation.
Hypothesis H5b: The level of materialism orientation moderates the relationship between CSR and expected pride such that the relationship is stronger for job applicants with a low materialistic orientation than for those with a high materialism orientation.

Based on the hypotheses we propose a moderated-mediation hypothesis, namely that the level of job seeker’ materialism orientation negatively moderate the indirect effect of CSR on job applicant via employer reputation. Specifically, a low level of materialism-orientation has a stronger indirect effect of CSR on job applicant attraction via employer reputation. In comparison, a high level of materialism orientation has a weaker indirect effect of CSR on job applicant attraction via employer reputation. Additionally, it is assumed that the level of materialism orientation negatively moderates the indirect effect of CSR on job applicant attraction via expected pride. Specifically, a low level of materialism orientation has a stronger indirect effect of CSR on job applicant attraction via expected pride. In comparison, a high level of materialism orientation has a weaker indirect effect of CSR on job applicant attraction via expected pride.

H6a: The indirect effect of CSR on job applicant attraction via employer reputation will be stronger when the level of materialism orientation is low rather than high.
H6b: The indirect effect of CSR on job applicant attraction via expected pride will be stronger when the level of materialism orientation is low rather than high.

**Fig 1:**
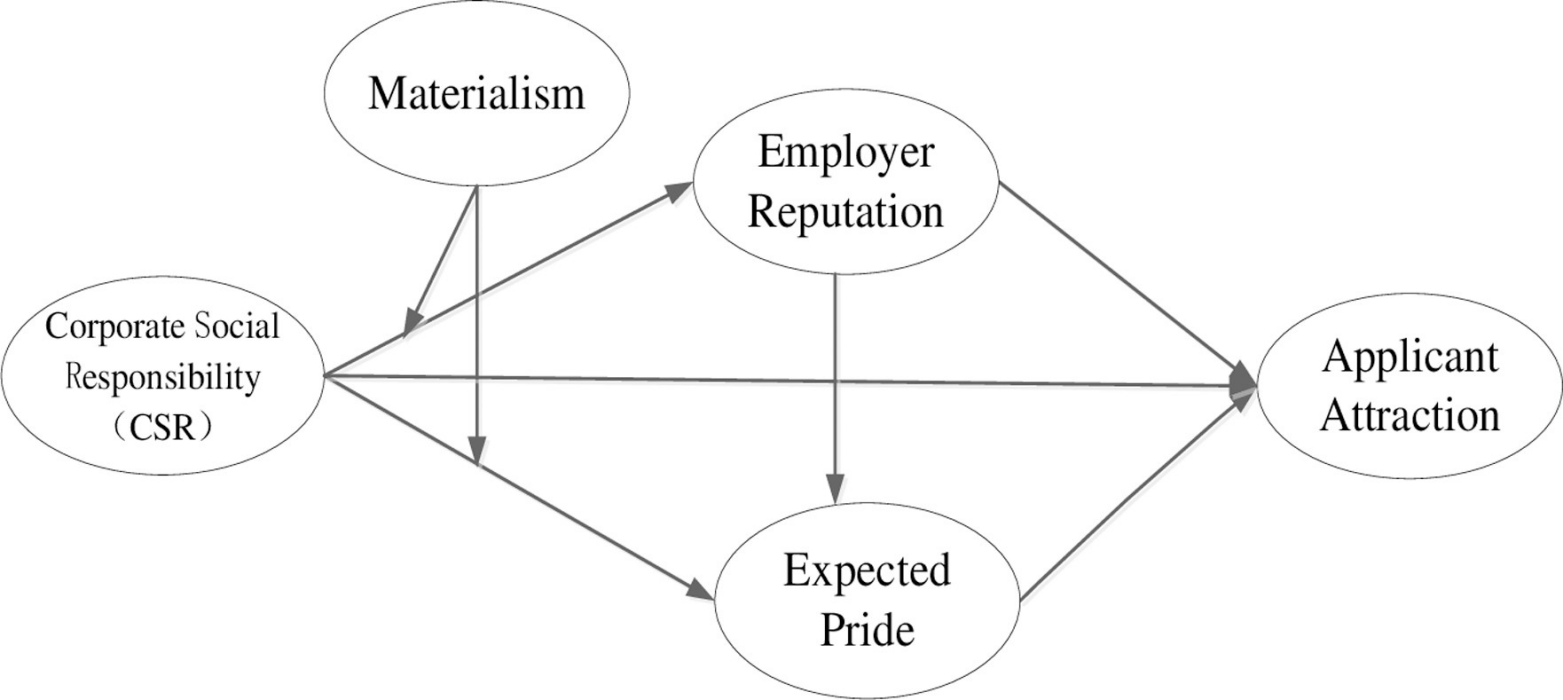
Theoretical framework.

Thus, based on the above theoretical foundations, we derived the theoretical framework for this research, as shown in Fig 1.

## Methodology

### Participants and procedure

This study consisted of 402 university students from Huazhong University of Science and Technology, located in central China. The participants were recruited on a voluntary basis through advertising on social media, and snowball recruitment was also utilized, wherein participants were encouraged to inform others about the study. All participants provided an informed and written consent for participation and were aware of taking part in the presented study. Additionally, to eliminate the influence of inconsistent organization popularity on its job applicant attraction, this study selects three well-known firms, namely A, B, and C, known for their CSR participation such as free educational institutes, shelter houses, medical facilities, left-behind children in rural communities. A is an electrical appliance manufacturing firm, and its products are famous all over the world; B is an e-commerce firm, and its business scope spreads all over the world; C is a communication technology firm that makes and sells communication equipment for worldwide users.

Studies on CSR have mainly collected data from questionnaire surveys. Recently, along with the cross-boundary integration of CSR with other themes, more and more scholars have begun to adopt experimental methods to explore CSR topics. The advantage of an experimental methodology that can effectively control the experimental process and affect factors for the benefit of scholars, eliminating interfering factors and clarifying the core causalities among variables, has been widely recognized by scholars. Therefore, this research, to focus on the causal relationships among CSR, job applicant attraction, employer reputation, expected pride and materialism, and exclude other affecting factors(such as enterprise-scale), uses a combination methodology of experiment and questionnaire to verify the theoretical hypotheses.

The experiment was carried out and completed in half an hour. The respondents were required to enter a recruitment room designed to simulate the real scenario of recruitment. The procedure was as follows:

First of all, the experimental participants were randomly assigned to the divided groups (the high-CSR group and the low-CSR group). The two groups respectively read the experimental materials related to basic information (business performance, and remuneration) about three firms of A, B, and C, except that the CSR information was different. Secondly, both groups were asked to rate the job attraction extent of the three firms, as well as the perceived levels of CSR practices. Thirdly, based on the analysis of the previous step, the experiment adopts a 2 (high-level CSR vs. low-level CSR) × 3 (Firm A vs. Firm B vs. Firm C) design to confirm the relationship between CSR and job applicant attraction. In line with the procedure in the first step, the participants read different materials about three firms to control the level of CSR. Participants in the high-CSR group were informed: firm A has spent 3 percent of its net profit on social welfare donation activities, and 3 percent of its net profit on improving the working environment of employees every year; firm B has spent 3 percent of its net profit on public welfare activities, and 3 percent of its net profit on improving the working environment of employees every year; environmental protection and community construction in firm C are estimated to have cost 5 percent of the net profit every year. Participants in the low-CSR group read the information that the proportion of the three firms’ investment in CSR activities was 0.3%, 0.3%, 0.3%, 0.3%, 0.5%, respectively. Both groups were required to read their respective materials and appraise the job attraction inspired by the three firms, as well as the perceived levels of CSR practiced by firms A, B, and C. The experiment was completed after the participants provided personal information.

In this experiment, 402 questionnaires were handed over to rate the CSR, employer reputation, expected pride, materialism, and job applicant attraction. In total, 395 (98%) usable questionnaires were received, including 201 valid samples from the high-CSR group and 194 samples from the low-CSR group. Of the 395 participants who responded, 73.7% were male, and the average age was 20.5 years. Respondents included seniors (54.7%), juniors (34.2%) and sophomores (12.1%). Approximately 78 percent of the respondents indicated that they were searching for a job. Moreover, most of the participants (63.4%) were majoring in science and technology, and 40.2% participants’ average annual household income (AAHI) ranged from 50,000 to 100,000 RMB.

### Measurement

All the variables were assessed using measures that have been frequently applied and validated in existing research. We followed the translation and back-translation procedure to validate the translation quality, and asked the respondents to rate the extent of CSR, employer reputation, expected pride and job applicant attraction using five-point Likert scales ranging from 1 (strongly disagree) to 5 (strongly agree).

#### Corporate social responsibility (CSR)

The four-dimensional structure (Seventeen items) of CSR provided by Turker was used to measure the job seeker’s attitude to the potential employer [41]. It reflected the responsibilities of a business to various stakeholders, such as social and nonsocial stakeholders, employees, customers, and government. The questionnaire had been used in the Chinese cultural context [11] and demonstrated idea reliability and validity. An example item is "I believe that the company is socially responsible." The Cronbach’s *a* was 0.948.

#### Employer reputation

Employer reputation questionnaire (four items), initially developed by Turban et al., was used to rate job seeker’ reputation perception of the potential employer [42]. An example item is "I have heard a lot of good things about this firm." The Cronbach’s *α* was 0.893.

#### Expected pride

Three measurement items of expected pride, developed by Turban et al., was to assess job seeker’s expected pride from future organizational membership [43]. An example item is "I would be proud to identify myself personally with this firm." The Cronbach’s *α* was 0.911.

#### Materialism

The measurement of materialism developed by Richins and Dawson was used in our study and included 18 items [24]. An example item is "My life would be better if I had what I do not have." The Cronbach’s *a* was 0.824.

#### Job applicant attraction

Following previous research [44], the present study assessed job applicants’ attraction to the organization on two dimensions perceived desirability(six items) and job pursuit(five items). An example item is "I would want this company to recruit on my campus." The Cronbach’s *a* was 0.896.

#### Control variables

Following previous studies (e.g., [44]), this study controlled for demographic variables such as job seekers’ gender, grade, major, and average annual household income (AAHI) that may influence job applicant attraction.

## Results

### Manipulation check

F-tests were used to compare baseline levels of job applicant attraction variables between the high-CSR group and the low-CSR group. F-tests were performed by SPSS 22.0 version and a *p* value of less than 0.05 was considered statistically significant, and effect size was measured by Cohen’s *d* using GPower 3.1. According to Cohen [45], effect size was interpreted as small (Cohen’s *d ≥* 0.20), medium (Cohen’s *d ≥* 0.50) and large (Cohen’s *d ≥* 0.80).

Table 1 showed that the CSR means of firms A, B, and C rated by the high-CSR group participants were all higher than the means of the low-CSR group’s perceptions. The high-CSR group participants’ total perception means of the CSR (*M* = 3.688, *SD* = 0.490) was also clearly higher than the low-CSR group’s total perception mean (*M* = 3.1587, *SD* = 0.432), and the mean comparison difference between the high-CSR group and the low-CSR group was statistically significant (*F* = 21.054, *p* < 0.001), which indicated that CSR was significant in this research.

**Table 1:** Mean and F-test of CSR ratings.

Company	Variable	The high-CSR group		The low-CSR group		*F*	*P*
		*M*	*SD*	*M*	*SD*		
**A (N = 85)**	CSR	3.312	0.116	2.889	0.221	7.090	0.045
**B (N = 78)**	CSR	3.664	0.102	3.226	0.116	8.002	0.043
**C (N = 90)**	CSR	4.164	0.556	3.556	0.074	28.288	0.000
**Total (N = 395)**	CSR	3.688	0.490	3.158	0.432	21.054	0.001

Note: *M* = mean, *SD* = the standard deviation,

*p < 0.05,

** *p* < 0.01.

**Fig 2:**
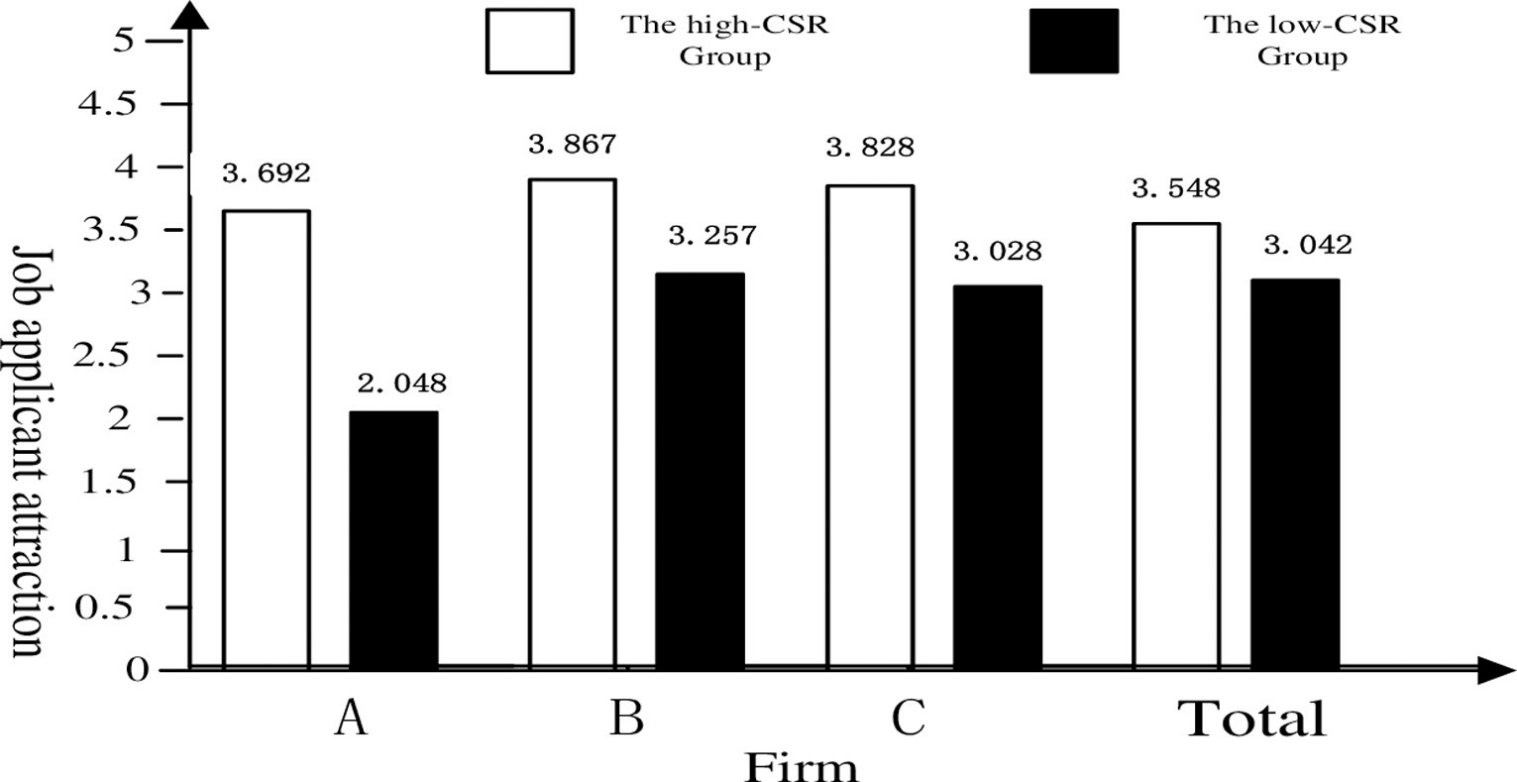
One-way analysis variance of CSR and job applicant attraction.

Fig 2 indicated that the rating means of job applicant attraction to firms A, B, and C from the high-CSR group was noticeably higher than the means of the low-CSR group’s evaluation. And the results also revealed that participants in the high-CSR group had a significantly higher level of job applicant attraction than those in the low-CSR group (3.548 ± 0.908 *vs* 3.042 ± 0.878, *F* = 12.171, *p* < 0.01, *d* = 0.566).

### Measurement validity and Common bias test

A Confirmatory factor analyses (CFA) results were evaluated using the *x*^*2*^*/df* statistic [46], and a variety of other fit indices (i.e., the root mean square error of approximation (RMSEA), the goodness-of-fit index (GFI), the normed fit index (NFI), the comparative fit index (CFI), and the incremental fit index (IFI)). As suggested by Schweizer [47], values for the RMSEA equal to or smaller than 0.08 were considered acceptable; values for the GFI, NFI, CFI and IFI equal to or greater than 0.90 were considered acceptable, while values close to 0.95 or higher were considered good. The five-factor model that included CSR, materialism, expected pride, employer reputation, and job applicant attraction was initially assessed (see Table 2), and the fit indices showed that this model was quite acceptable (*x*^*2*^*/df* = 1.653, CFI = 0.939, NFI = 0.912, GFI = 0.902, RMSEA = 0.052). Subsequently, Cmmon-method bias was assessed with Harman’s Single-Factor Test [48]. A sequential *χ*^*2*^ difference test showed that the one-factor model was significantly poorer than the five-factor model (*Δχ*^*2*^(28) = 2697.97, *p* < 0.01), which indicated that common method bias was not a critical issue.

**Table 2:** Model fit summary for hypothetical model and alternative models.

Model	*X* ^*2*^ */df*	CFI	NFI	GFI	RMSEA
**Hypothetical five-factor model** CSR, M, EP, ER, JAA	1.653	0.939	0.912	0.902	0.052
**Alternative four-factor model** CSR+ER = 1 factor, M, EP, JAA	1.817	0.922	0.843	0.887	0.058
**Alternative three-factor model** CSR+EP+ER = 1 factor, M, JAA	3.428	0.764	0.700	0.762	0.100
**Alternative two-factor model** CSR+M+EP+ER = 1 factor, JAA	3.831	0.722	0.661	0.740	0.108
**Alternative one-factor model** CSR+M+EP+ER+JAA = 1 factor	4.881	0.616	0.565	0.653	0.127

Note:CSR = corporate social responsibility; M = materialism; EP = expected pride; ER = employer reputation; JAA = job applicant attraction.

### Statistics analyses

Means(*M*), standard deviation (*SD*), and correlations regarding the study variables are displayed in Table 3. Descriptive analyses with SPSS 22.0 version were based on *M* and *SD*. Preliminary analyses were carried out to test the relationships between demographic variables (gender, grade, major, and AAHI), predictors (CSR), and outcome variables (Job applicant attraction). In the analysis, grade and AAHI were entered as continuous variables, and the categorical variables were coded as follows: gender (male  =  0, female  =  1), major (science majors = 0, Non-science majors = 1). Independent-samples T test result indicated that the participants’ demographic characteristics (gender, grade, major, and AAHI) did not affect their perceptions of job applicant attraction. Pearson’s correlation analysis was used to explore the bivariate association between measured variables, and a *P* value < 0.05 was defined as the level of significance. Table 3 showd CSR was found to be significantly related to the the mediator, namely expected pride(*r* = 0.37, *p* < 0.01), employer reputation(*r* = 0.66, *p* < 0.01). And CSR was positively related to the dependent variables, i.e., job applicant attraction(*r* = 0.28, *p* < 0.01). Employer reputation was also found to be linked to expected pride(*r* = 0.49, *p* < 0.01).

**Table 3:** Descriptive statistics and correlations among variables.

Variables	*M*	*SD*	1	2	3	4	5	6	7	8	9
gender	1.19	0.51	--								
Grade	1.60	0.78	-0.02	--							
Major	2.53	0.68	-0.31**	-0.09	--						
AAHI	2.88	1.33	-0.02	0.13*	-0.12	--					
CSR	3.42	0.53	-0.07	-0.08	0.07	-0.10	--				
M	3.20	0.74	-0.01	0.09	0.06	-0.02	0.31**	--			
EP	3.47	0.86	0.02	-0.10	-0.03	-0.07	0.37**	0.23**	--		
ER	3.48	0.60	0.06	-0.04	0.01	-0.06	0.66**	0.27**	0.49**	--	
JAA	3.44	0.88	0.04	-0.13	-0.04	-0.09	0.28**	0.25**	0.60**	0.41**	--

Note: N = 395,

*p < 0.05,

**p < 0.01. AAHI = average annual household income; CSR = corporate social responsibility; M = materialism; EP = expected pride; ER = employer reputation; JAA = job applicant attraction.

### Hypotheses testing

The mediating results of the three-stage hierarchical regression analysis [49] were presented in Table 4. Following Sinacore, all independent and mediating variables included in the study were mean-centered to deal with multicollinearity [50]. In step 1, the control variables were entered into the model: they explained 1.6 percent of the variance in expected pride (Model 1), 0.6 percent in employer reputation (Model 3), and 1.3 percent in job applicant attraction (Model 5). In step 2, the independent variable was added to the regression. The results showed that CSR positively affects job applicant attraction (Model 6, *β* = 0.672, *p* < 0.01), Hypothesis 1 was supported.

**Table 4:** Hierarchical regression analysis of the mediating effect.

Variables	Expected pride		Employer reputation		Job applicant Attraction						
	M1	M2	M3	M4	M5	M6	M7	M8	M9	M10	M11
gender	0.021	0.042	0.037	0.031	0.041	0.037	0.009	0.012	0.026	0.037	0.007
Grade	-0.090	-0.065	-0.090	0.016	-0.003	0.017	0.051	0.053	0.008	0.009	0.047
Major	-0.022	-0.043	-0.022	-0.019	-0.038	-0.053	-0.024	-0.028	-0.045	-0.046	-0.027
AAHI	-0.056	-0.028	-0.056	-0.003	-0.096	-0.074	-0.062	-0.058	-0.073	-0.073	-0.059
CSR		0.360**		0.657**		0.672**		***0*.*065***		***0*.*014***	**-0.029**
EP							0.602**	0.579**			0.531**
ER									0.405**	0.396**	0.169*
*R* ^*2*^	0.016	0.145	0.006	0.430	0.013	0.087	0.370	0.373	0.176	0.176	0.388
*ΔR* ^*2*^	0.000	0.127	-0.011	0.418	-0.003	0.068	0.357	0.358	0.159	0.155	0.369
*F*	0.998	8.028**	0.354	35.805**	0.789	4.510**	27.824**	23.447**	10.123**	8.407**	21.258**

Note: N = 395,

*p < 0.05,

**p < 0.01. AAHI = average annual household income; CSR = corporate social responsibility; EP = expected pride; ER = employer reputation.

Meanwhile in step 2, the effect of CSR on expected pride (Model 2, *β* = 0.360, *p* < 0.01) and employer reputation (Model 4, *β* = 0.657, *p* < 0.01) was positive. In step 3, the independent and mediating variables were added to the model. The results showed that expected pride (Model 7, *β* = 0.602, *p* < 0.01) and employer reputation (Model 9, *β* = 0.405, *p* < 0.01) had a significant effect on job applicant attraction. Independent variables and mediating variables were then entered into the regression. It can be seen that CSR was not significantly related to job applicant attraction (Model 8, *β* = 0.065, ns). However, a positive effect of expected pride on job applicant attraction was still significant (Model 8, *β* = 0.579, *p* < 0.01), The adjusted *R*^*2*^ accounted for approximately 37.3 percent of expected pride in job applicant attraction. Similarly, CSR had no significant effect on job applicant attraction (Model 10, *β* = 0.014, *ns*), but expected pride was still significantly related to job applicant attraction (Model 10, *β* = 0.396, *p* < 0.01) and explained 17.6 percent of job applicant attraction. Furthermore, both the independent variables and mediating variables were added to the model. In Model 11, CSR was uncorrelated with job applicant attraction (*β* = -0.029, *ns*), but expected pride and employer reputation still positively affected job applicant attraction (*β* = 0.531, *p* < 0.01; *β* = 0.169, *p* < 0.05). The findings showed that the employer reputation and expected pride mediated the relationship between CSR and job applicant attraction.

Furthermore, for testing mediating effects, the method of the bias-corrected bootstrap provides the most accurate confidence interval (CI) estimation and has the highest statistical efficacy [51]. Therefore, in the current study, a bootstrapping analysis was conducted using the SPSS macro PROCESS (with CSR as the independent variable, job applicant attraction as the outcome variable, employer reputation and expected pride as mediators, and gender, grade, major, and AAHI as covariates) with 5000 resamples to test a serial mediation model, and to calculate the 95% confidence interval(CI). The indirect effect was considered statistically significant if the 95% bias-corrected CI did not contain zero [52]. The direct and indirect effects of employer reputation and expected pride in the relationship between CSR and job applicant attraction were shown in Table 5. The indirect effect of CSR on job applicant attraction through employer reputation was significant (*β* = 0.266, *SE* = 0.11, 95% CI = [0.157, 0.375], excludes zero), the mediation effect (CSR → employer reputation → job applicant attraction) accounted for 39.525% of the total effect, hypothesis 2 was supported. Also, expected pride mediated the relationship between CSR on job applicant attraction (*β* = 0.216, *SE* = 0.13, 95% CI = [0.109, 0.323], excludes zero), the mediation effect (CSR →expected pride → job applicant attraction) accounted for 32.095% of the total effect, hypothesis 3 was supported. Finally, the indirect effect of CSR on job applicant attraction through employer reputation and then expected pride (i.e., a serial mediating effect) was also found (*β* = 0.191, *SE* = 0.06, 95%CI = [0.009, 0.373], excludes zero), the mediation effect (CSR → employer reputation → expected pride → job applicant attraction) accounted for 28.38% of the total effect, hypothesis 4 was supported.

**Table 5:** The bootstrapping analysis of the serial mediating effect.

Model pathways	Estimated effect *(β)*	95% CI	
		Lower	Upper
**Direct effects**			
CSR→JPA	0.672***	0.645	0.699
**Indirect effects**			
CSR→ER→ JPA	0.266**	0.157	0.375
CSR→EP→ JPA	0.216**	0.109	0.323
CSR→ER→EP→ JPA	0.191**	0.009	0.373
**Total effect**	0.673***	0.604	0.742

Note: N = 395,

**p < 0.01,

***p < 0.001. CSR = corporate social responsibility; EP = expected pride; ER = employer reputation; JAP = Job applicant attraction.

The moderating effect of materialism in the relationship between CSR and job applicant attraction was tested (see Table 6) using hierarchical regression analysis [49]. Following Sinacore, all independent and moderating variables included in the study were mean-centered to deal with multicollinearity [50]. In Model 2, CSR had a positive effect on expected pride (*β* = 0.315, *p* < 0.05). When both CSR and expected pride were entered into the regression (Model 3), the effect of CSR on expected pride was still significant (*β* = 0.317, *p* < 0.05), the CSR*materialism interaction was statistically significant for expected pride in Model 3 (*β* = -0.141, *p* < 0.05), accounting for 18.4% of the total variance. The result indicated that materialism played a negative moderating effect on the relationship between CSR and expected pride. Hypothesis 5b was supported. Moreover, the PROCESS macro with 5000 resamples was used to generate bootstrap confidence intervals for the conditional indirect effect of the CSR on job applicant attraction via expected pride at different levels of job seekers’ level of materialism orientation. When job applicants with a low level of materialism orientation, CSR have a significant indirect effect on job applicant attraction through expected pride (*b* = 0.202, *SE* = 0.08, 95%CI = [0.11, 0.29], excludes zero), however, it turned into insignificant (*b* = 0.02, *SE* = 0.01, 95%CI = [−0.01, 0.03], contains zero) while job seekers’ level of materialism orientation was high. And the pairwise contrasts between these conditional indirect effects was significant (*b* = 0.008, *SE* = 0.03, 95%CI = [0.002, 0.014], excludes zero). Consequently, Hypothesis 6b was supported.

**Table 6:** Hierarchical regression analysis of the moderating effect.

Variables	Expected pride			Employer reputation		
	M1	M2	M3	M4	M5	M6
**gender**	0.021	0.049	0.048	0.038	0.029	0.029
**Grade**	-0.090	-0.083	-0.083	-0.029	0.006	0.006
**Major**	-0.022	-0.049	-0.051	0.018	-0.022	-0.023
**AAHI**	-0.056	-0.028	-0.032	-0.055	-0.004	-0.005
**CSR**		0.315*	0.317*		0.632*	0.633*
**M**		0.148*	0.168*		**0.080**	**0.088**
CSR*M			***-0*.*141*** *			***-0*.*056***
***R*** ^***2***^	0.016	0.164	0.184	0.006	0.436	0.439
***ΔR*** ^***2***^	0.000	0.062	0.060	0.002	0.196	0.158
***F***	0.998	7.731*	7.559*	0.354	30.410*	26.284*

Note: N = 395,

*p < 0.05,

** *p* < 0.01,

*** *p* < 0.001. AAHI = average annual household income; CSR = corporate social responsibility; M = materialism.

Meanwhile, Model 5 showed that the link between CSR and employer reputation was significant (*β* = 0.632, *p* < 0.05). When both CSR and employer reputation were entered into Model 6, the result indicates that CSR was positively correlated with employer reputation (*β* = 0.633, *p* < 0.05), but the CSR*materialism interaction was not statistically significant for employer reputation (*β* = -0.056, *ns*). Hypothesis 5a was not supported. Moreover, the PROCESS macro with 5000 resamples was used to generate bootstrap confidence intervals for the conditional indirect effect of the CSR on job applicant attraction via employer reputation at different levels of job seekers’ level of materialism orientation. The pairwise contrasts between these conditional indirect effects was not significant (*b* = 0.011, *SE* = 0.02, 95%CI *=* [-0.003, 0.025], includes zero). Hypothesis 6a was not supported.

**Fig 3:**
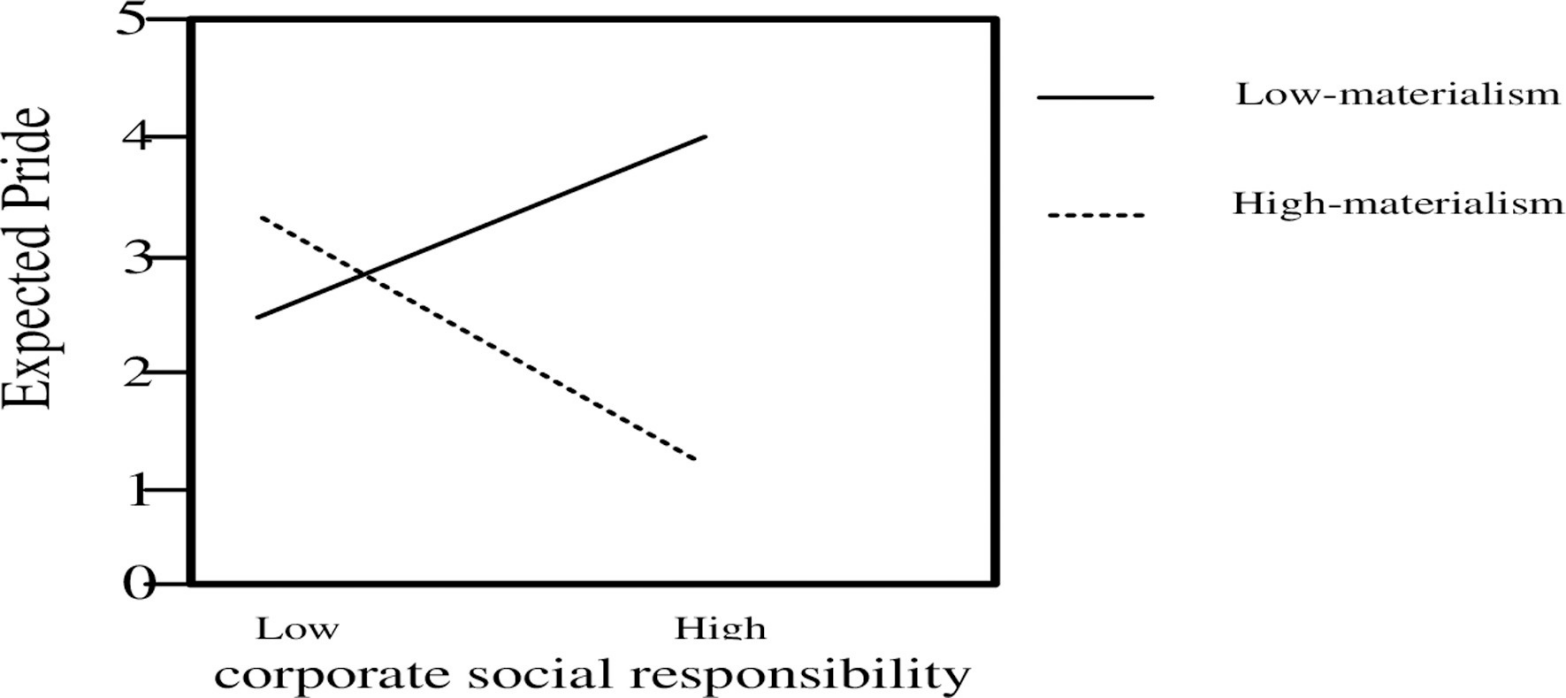
Moderating effect of materialism on the relationship between CSR and expected pride.

To further investigate the moderating effects of individual materialism orientation in greater detail, regression equations were plotted at different levels of materialism (i.e., one standard deviation above and below their mean; see Sinacore [50]). The results of the equations were presented graphically in Fig 3. As shown, the positive relationship between CSR and expected pride was weaker as the level of their materialism orientation increased, and jobseekers with a high level of materialism orientation exhibited lower levels of expected pride, regardless of the level of CSR activity.

## Discussion

### Main findings and theoretical contributions

The current analysis highlights the main findings, and three significant contributions related to results are introduced. Our study confirms that CSR is positively related to job applicant attraction, following the conclusions of most studies on the positive impact of CSR on job applicant attraction [7,8,53]. Additionally, the result validates the mediation effect of employer reputation and expected pride on the relationship between CSR and job applicant attraction, respectively, and the serial mediating role of employer reputation and then expected pride in the relationship between CSR and job applicant attraction. Furthermore, the findings indicate that job applicants’ materialism orientation plays a moderating role in the indirect effect of CSR on job applicant attraction via expected pride, but the moderating effect of job seekers’ materialism orientation in the indirect effect of CSR on job applicant attraction via employer reputation is not statistically significant.

The first theoretical contribution is to explain CSR’ positive relationship with job applicant attraction from the Chinese traditional Confucian cultural perspective. Confucian humaneness values advocate people’s social obligations and ethical decision-making [13,14]. CSR activities beyond the immediate organization’ and its shareholders’ interests are the best expression of humaneness view of Confucian ethics. Thus the positive CSR activities have a demonstrably more substantial impact on the Chinese job seeker’s intentions toward the potential employer. However, Hofman and Newman propose that Chinese employees will respond less positively to CSR practices than employees in the West due to decreased job security levels and high levels of in-group collectivism [11], which seems inconsistent with our results. We consider the difference in the sample may cause this. The sample used by Hofman and Newman’s mainly contains production workers and shop floor managerial employees at lower levels of the organization [11], who have limited influence on the employer’s strategy and interaction with external stakeholders. The characteristics of millennial-aged job seekers in our sample are owning good education [8,46], practicing moral behavior guided by Confucius [16], and placing the most importance on intrinsic and social aspects of work [15]. Additionally, we indicate those job seekers also influenced by a collectivist culture will feel a status identity so that they may be pride in their organization’s CSR practices. These results put forward a new culture-driven interpretation of (potential) employees’ responses to CSR.

The second contribution is to explore the mediating effect of employer reputation and expected pride on the relationship between CSR and job applicant attraction based on social identity theory, which responses to the lack of study of the psychological mechanism of CSR affecting job applicant attraction [6] and provides new ideas for further clarifying the ’black box’ of CSR’s role in job applicant attraction. Additionaly, we have found support for the serial mediation model. CSR is indirectly associated with job applicant attraction through employer reputation and then expected pride. Similarly, prior studies have consistently demonstrated that reputation can positively influence the pride individuals expect from organizational membership, and subsequently affect job pursuit and organizational behavior [30,35]. Social identity theory presents that individuals identify with their groups through social classification and maintain a positive emotion with the organization they identity [5,36]. Job applicants’ employment intentions are the social identification process through various information signals from the prospective employer. When job seekers believe that prospective employers engage in CSR activities beyond what average organization can do [5,17], they tend to attribute a favorable reputation to the employer [5,7,33] and anticipate experiencing if they are associated with the organization as one of its members [5]. Meanwhile, the feelings of pride in being in-group members of a well-respected and well-regarded organization make the organization more attractive to job applicants [6,28]. In conclusion, the psychological mechanism of CSR in job applicant attraction is formed mainly through the social identification mechanism.

The third contribution of this research highlights the moderating role of job applicants’ materialism orientation in determining their attitudinal response to CSR, especially expected pride perception. This theme is the first to apply individual materialism values in the recruitment field and serves to expand the previous results focusing on its discussion in the personal consumption and values field [20,21,23]. The finding that individuals’ materialism orientation negatively moderates CSR’ correlation with expected pride suggests that job applicants scoring high in materialism emphasize material acquisition no matter how well the company performs its social responsibility. Job seekers with a high materialism orientation believe that material possessions are more substantial than the positive emotional experience brought about by CSR practice. Therefore, CSR’ positive influence on the job applicants’ perception of expected pride significantly weakens, which in turn reduces their willingness to pursue jobs. Conversely, individuals scoring low in materialism have higher hopes and expectations of spirited possessions, and it may be easier for them to be satisfied with what they have compared to others; these job seekers may, therefore, emphasize on the pride generated by an employer’s CSR fulfillment, which in turn enhances the job application desire. This result is also consistent with the Chinese economic situation, characterized by the accelerated accumulation of wealth, which has extensively promoted the rise of materialism [22], individuals become more interested in maximizing their welfare [11]. However, interestingly, the result, in contrast to what was hypothesized, indicates that the high or low materialism values of job applicants are insignificant for positive perceptions of CSR—employer reputation. We believe that the reason for this finding may be the hidden meaning of materialism. Although the utilitarian tendency of a materialist who has higher hopes and expectations of material possessions is evident, he or she is also a prudent and informed investor focusing on long-term returns as well as short-term acquisition. In this regard, employer reputation generated by CSR is not only an intangible asset for the employer, but also in the long term brings benefits for job seekers who may gain material acquisitions and success based on the employer’s good reputation, in keeping with materialism. In this context, job seekers, instead of short-term interests, will grasp the possible opportunity to acquire long-term material possessions, and they may deliberately hide their materialistic tendency. Of course, there may be other factors driving this conclusion that can be considered in future studies.

### Management implications

The conclusions of this study have significant management implications for talent attraction. On the one hand, the findings indicate that CSR practices’ development and implementation is one human resource strategy utilized by organizations to enhance the attraction of qualified talent. The organization should leverage various social media to show job seekers its CSR activities [54]. In particular, when job seekers are unfamiliar with the prospective employer, or they face a comparative choice among employers at the same level, CSR practice becomes an important signal which is helpful for their decisions and behaviors. CSR activities not only target internal stakeholders (such as training, welfare, work environment) but also focus on external stakeholders (such as green production, environment, public welfare support). Among these activities, one important aspect of CSR towards society in Chinese firms is philanthropic donations [11], despite widespread evidence show that corporate philanthropy boosts corporate growth and profitability, these ultimate controllers indicate no intention to donate their own money as a means to improve corporate performance [55]. On the other hand, managers need to realize that the relationship between job applicants’ response to CSR activities and job attraction partly depends on an individual’s materialism values. Therefore, when designing CSR policies, managers should consider the different extent of individuals’ materialism values. For example, recruitment information about CSR policies aimed at internal stakeholders may attract the job applicant scoring high for materialism, and the CSR practice targeted at external stakeholders may be more attractive to the low-materialism individual. Furthermore, the organization should develop other strategies to satisfy job applicants’ material needs according to the development stage of organizations [11], such as offering competitive payment, establishing a performance-oriented and ability-oriented compensation system, and so on.

### Limitations and future research

This study is not without limitations. Firstly, although some scholars believe that a study from a single-level perspective is more predictive than that from a cross-level perspective [56], the methodology of self-report and the signaling perspective applied in this study may cause CSR perception biases, so future research should consider adapting the methodology of self-report and report by others to collect CSR data. Secondly, this research has verified the positive effect of CSR on job applicant attraction. Not all organizations consider CSR activities as an obligation, so on the other side of the coin, the idea that organizations failing to fulfill social responsibilities may bring a negative emotional experience to job seekers should be addressed in the future. Alongside confirmation of the mediating role of expected pride and employer reputation in the relationship between CSR and job applicant attraction, future research needs to explore the effect mechanism of the other possible psychological variables, such as social identity, self-esteem. Thirdly, the findings indicate that the individual’s materialism moderates the relationship between CSR and job applicant attraction. However, job applicants’ perceptions are changeable because of social development, especially the information found in this survey that post-1990s job seekers pay more attention to their career interests and prefer entrepreneurial organizations. Therefore, future research on job applicant attraction should discuss from multiple perspectives.

## Supporting information

S1 Dataset (ZSAV)
